# Hay Fever

**Published:** 1903-04-25

**Authors:** 


### HAY FEVER.

Sufferers from hay fever will now be rejoicing, no doubt, that their special malady, which has hitherto made them look forward with something like dread to the approach of early summer, is at this moment the subject of extensive experimental research at the hands of Professor Dunbar, of Hamburg, and that so far as his investigations go, judging from Sir Felix Semon's report in the *British Medical Journal* of March 28th and April 18th, there seems to be a reasonable likelihood that in the near future medical men will be in a position to treat the complaint on a basis of rational pathology and therapeutics. Up to the present time, the only sure way of obtaining relief from the affection has been to run away from it, and to abide the summer in some place where the baleful pollen is not, a course obviously impossible for the many. Now, however, according to Professor Dunbar, the toxin has been discovered. He has isolated from the pollen of certain grasses a toxic substance which, when applied in very dilute solution to the eyes or nostrils of individuals who are subject to hay fever, produces within a few minutes the characteristic local symptoms of the complaint. The toxin solution has no effect upon one who is not a sufferer from hay fever. By injecting the pollen of various grasses into the circulation of certain animals—rabbits, goats and horses—Professor Dunbar has succeeded, he believes, in producing an active antitoxin, which, when applied to the eyes and nostrils of hay-fever patients in whom the local symptoms have been produced artificially by the previous employment of the toxin, immediately quells the subjective symptoms, and in a few minutes causes the objective signs to subside. Grasses appear to be the chief offenders in causing the affection, for Professor Dunbar has failed to discover the toxin in the pollen of roses and many other flowers. The toxin was obtained in sufficient quantity for experimental purposes by extracting the crushed pollen of maize with saline solutions at the body temperature for about six hours, and by precipitating the toxin from the solution with alcohol. The antitoxin was obtained by injecting the toxin into horses. A full report of Professor Dunbar's investigations will be welcomed with a considerable amount of interest by the medical profession at large.

